# Wake respirometry allows breath-by-breath assessment of ventilation and CO_2_ production in unrestrained animals

**DOI:** 10.1016/j.isci.2022.104878

**Published:** 2022-08-14

**Authors:** Kayleigh A.R. Rose, Rory P. Wilson, Claudia Ramenda, Hermina Robotka, Martin Wikelski, Emily L.C. Shepard

**Affiliations:** 1 Biosciences, Swansea University, Singleton Park, Swansea, UK; 2 Max Planck Institute of Animal Behaviour, Radolfzell, Germany; 3 Max Planck Institute of Animal Behavior, Radolfzell, Germany; 4 Centre for the Advanced Study of Collective Behavior, University of Konstanz, Konstanz, Germany

**Keywords:** Wildlife behavior, Physiology, Animal physiology, Methodology in biological sciences

## Abstract

Quantifying stress and energetic responses in animals are major challenges, as existing methods lack temporal resolution and elevate animal stress. We propose “wake respirometry,” a new method of quantifying fine-scale changes in CO_2_ production in unrestrained animals, using a nondispersive infrared CO_2_ sensor positioned downwind of the animal, i.e., in its wake. We parameterize the dispersion of CO_2_ in wakes using known CO_2_ flow rates and wind speeds. Tests with three bird species in a wind tunnel demonstrated that the system can resolve breath-by-breath changes in CO_2_ concentration, with clear exhalation signatures increasing in period and integral with body size. Changes in physiological state were detectable following handling, flight, and exposure to a perceived threat. We discuss the potential of wake respirometry to quantify stress and respiratory patterns in wild animals and provide suggestions for estimating behavior-specific metabolic rates via full integration of CO_2_ production across the wake.

## Highlights

• We use open-path nondispersive infrared spectroscopy CO_2_ sensor technology
• We measure ventilation rate and CO_2_ production in the wake of unrestrained animals
• Rapid responses to stressors and recovery from exercise can be measured
• Metabolic rate could be calculated by full integration of the wake of exhaled CO_2_

## Graphical abstract

**Figure:**
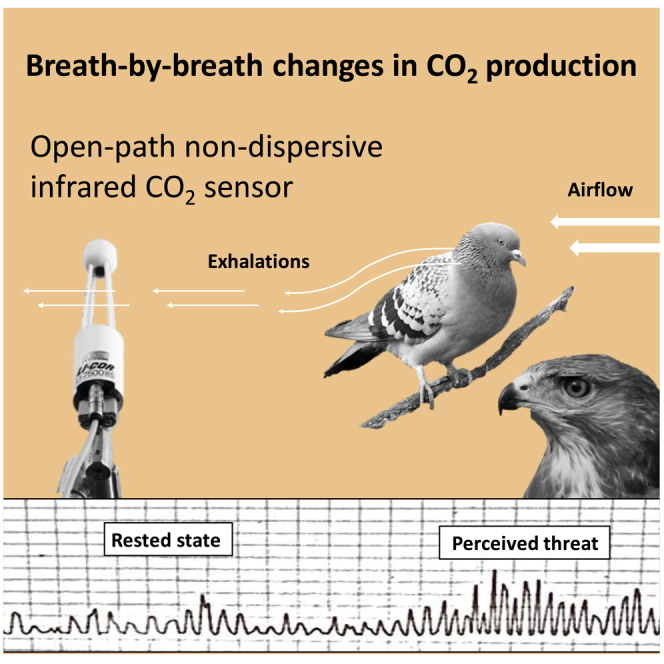

## Introduction

Determination of the energy expenditure of animals is pivotal for understanding the costs and rewards of behaviors and elucidating strategies that enhance lifetime reproductive success (Lemon, 1991; Shaffer et al., 2003). However, quantification of behavior-specific costs is a major challenge, both in the lab and field, due to ethical and technical drawbacks (Wilson and Culik, 1993; Butler et al., 2004). The gold-standard method, respirometry, is most often conducted in the lab and works by assessing the rate of CO_2_ production and/or O_2_ consumption (Lighton, 2019). A limitation of respirometry is that animals must either be confined to boxes (Hawkins et al., 2000) or equipped with masks (Ward et al., 2001; Morris et al., 2010; Langman et al., 2012), which prohibit or constrain expression of movement-related behaviors and can induce stress in the study animal (Tucker, 1972). Methods that have provided key insight in freely moving animals include doubly labeled water: injecting isotopes and later drawing blood samples to assess the rate of CO_2_ production (Nolet et al., 1992; Bevan et al., 1995a; Elliott et al., 2013) and either implanting or externally attaching loggers to measure real-time proxies for energy expenditure including heart rate (Nolet et al., 1992; Bevan et al., 1994, Bevan et al., 1995a; Ward et al., 2002; Green, 2011) and dynamic body acceleration (Wilson et al., 2006, 2020; Halsey et al., 2009, 2011). All require animal capture (and often recapture), known to cause a stress-response. Furthermore, doubly labeled water is invasive and cannot provide activity-specific information (Nagy et al., 1984; Wilson and Culik, 1993), although judicious use of animal-attached tags is providing a way forward (Sutton et al., 2021). Furthermore, proxies for energy expenditure first require calibration in the lab with either respirometry (Halsey et al., 2009, 2011; Gleiss et al., 2011; Halsey and Bryce, 2021) or doubly labeled water (Nolet et al., 1992; Bevan et al., 1995b; Elliott et al., 2013). Accelerometry is the only method that can provide sub-second behavior-specific data for free-living animals, but this only works if animals are moving and cannot provide information on changes in internal state, e.g., during thermoregulation, brooding, or stress (Wilson et al., 2020).

**Figure 1:**
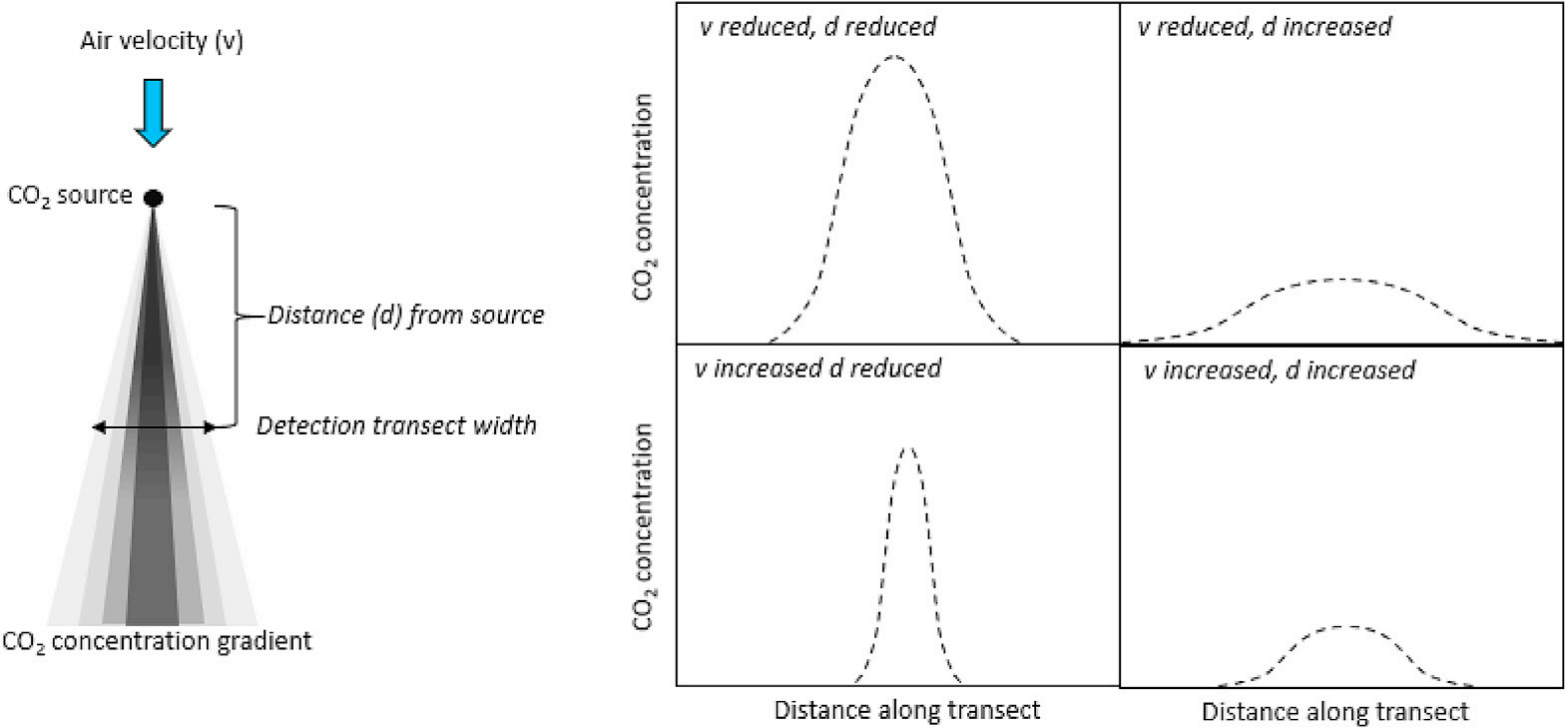
Expected changes in CO_2_ concentration in relation to sensor distance from the source and wind speed The distribution of CO_2_ emitted at a constant rate from a point source in space is roughly expected to follow an increasing radius of the iso-concentrations as the gas diffuses out, modulated by air flow, which will tend to distribute the CO_2_ downwind of the source, with distance-dependent iso-concentration radii decreasing with increasing air speed.

During the years that these processes have been developed and refined, our ability to determine CO_2_ concentration with high accuracy, even at low concentrations, has advanced dramatically. In particular, nondispersive infrared spectroscopy (NoDIS) has been shown to resolve CO_2_ concentrations as low as 0.01 ppm (https://www.licor.com/env/products/gas_analysis/LI-7000/specifications.html). Exhaling (air-breathing) animals have CO_2_ concentrations around 4% when their respiratory gases leave their body, with this concentration being diluted with distance from the source due to diffusion and wind. Nonetheless, the accuracy of NoDIS means that the CO_2_ signal should be detectable at some distance from the CO_2_ source using a system that does not interact with the study animal in any physical way. We propose that it might be possible to assess breath-by-breath information on animal state, by positioning these NoDIS sensors close to unrestrained, and possibly even free-living, animals and examining CO_2_ concentrations over time, provided there is directional flow of air. This would necessitate mapping out a full 2-dimensional (2D) cross-section of the CO_2_ wake. We term this approach “wake respirometry” because the CO_2_ signal from an animal is drifted downwind and over the sensor. The concentration of CO_2_ at any point around an animal, and therefore the ability of a NoDIS to quantify it, will depend on the concentration of CO_2_ being emitted in the exhaled air, the position of the sensor relative to the source, the speed and direction of the wind passing over the animal (Figure 1), as well as the expiratory flow rate, and the rate of diffusion and dilution of CO_2_ in air and water vapor.

In this work, we take a first step toward this goal by describing the use of the NoDIS method in wind tunnels behind perched captive, but unrestrained, pigeons *Columba livia domestica*, a starling *Sturnus vulgaris*, and a zebra finch *Taeniopygia guttata*. Our aims are to (1) demonstrate detection of a CO_2_ signal downwind of unrestrained animals and (2) examine how this signal is affected by rate of CO_2_ emission, source-sensor distance, lateral position across the wake, and wind speed. We also (3) examine what the approach can tell us about animal state and respiratory physiology in perched pigeons post-flight, post-handling, and in response to a perceived threat. Finally, (4) we map out future directions for the method to integrate the full CO_2_ shadow downwind of a resting animal and even behind a bird flying in a wind tunnel to derive figures for metabolic rates from animals undertaking activities that are currently assessed using conventional means, with associated limitations.

## Results

### Definition of the CO_2_ wake downwind of a source

**Figure 2:**
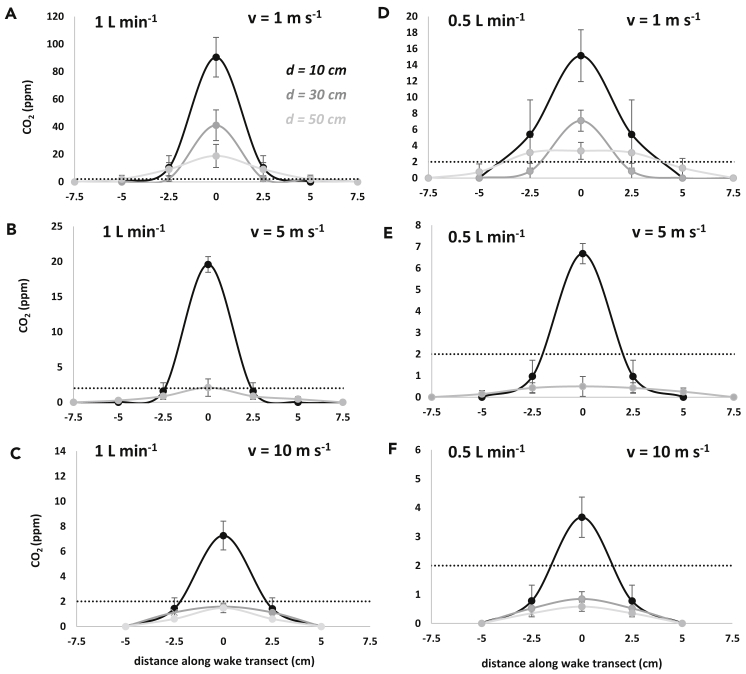
CO_2_ concentrations (mean ± SD) measured across the wake at different distances (d) from the source and windspeeds (v) (A–C) With a 4% CO_2_/air mix emission rate of 1 L min^−1^. (D–F) With an emission rate of 0.5 L min^−1^. The dotted line shows the position of the 2 ppm CO_2_ concentration. N.B data were collected at −7.5, −5, 0, 2.5, 5, and 7.5 cm from the midline. At −2.5 cm means ± SD mirror those on the opposite side of the wake.

We used a defined gas mix of 4% CO_2_/air (BOC) to carry out our calibrations in an open jet style wind tunnel custom designed for bird flight (test section width 1.8 m, length 2.2 m, height 1.5 m) in Swansea University, UK. Across a range of gas emission rates (0.5 L min^−1^ and 1 L min^−1^), source-sensor distances (10, 30 and 50 cm), and windspeeds (1, 5 and 10 m s^−1^), the width of the CO_2_ wake was measured using a NoDIS, LI-7500A Open Path CO_2_/H_2_O Analyzer (Lincoln, Nebraska, USA) sampling at 20 Hz and ranged from 5 to 14 cm (Figure 2). CO_2_ concentrations increased from the periphery of the wake toward a maximum in the center and decreased with increasing distance from the source and increasing windspeed (Figure 2). Transects across the downwind wake of a constant CO_2_ source showed that transect width increased with increasing distance from the source, apart from at the greatest windspeed of 10 m s^−1^ where the transect width remained narrow (Figure 2).

Using limits of detectable CO_2_ concentrations (here, 2 ppm) to define potential operational areas indicated that at a gas emission rate of 1 L min^−1^, the NoDIS sensor detected a clear CO_2_ signal up to 50 and 10 cm from the source at wind speeds of 1 and 10 m s^−1^, respectively (Figures 2A–2C). The same was found when the emission rate was halved (0.5 L min^−1^) (Figures 2D–2F).

### Bird CO_2_ wake exhalation signatures

**Figure 3:**
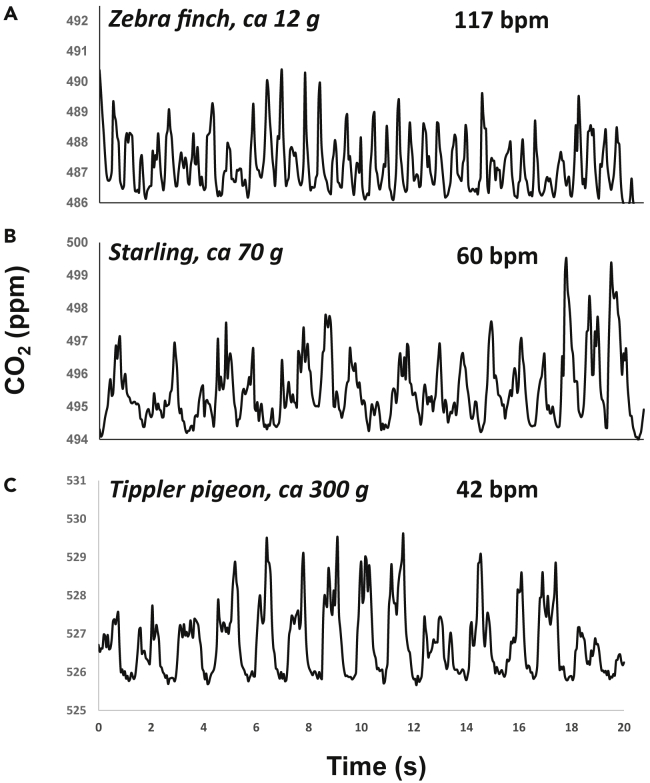
Raw CO_2_ exhalation signatures of three bird species of different body mass Body mass (g) and breathing frequency (breaths per minute) are also included.

In a closed system wind tunnel at the Max Planck Institute for Ornithology, Germany, we positioned the sensor 46 cm behind a zebra finch, starling, and pigeon with the windspeed set to 2 m s^−1^. Clear and regular peaks in CO_2_ were detectable for all three (Figure 3). These signals allow calculation of breathing frequency and the integral under the signal. However, as indicated by the calibration work, the quality of the signal depends on the rate of CO_2_ emission, and smaller species had less consistency in their peaks (Figure 3). The signal was clearest when the infrared path was aligned with the tail and body (as opposed to the head), indicating that the exhaled CO_2_ attached to the body. Some variation in the signal amplitude is expected due to movement of the head, which would influence integral calculations, but birds moved their heads less with the tunnel air turned on. Measures of breathing frequency should not be affected by head movement unless an exhalation is directed away from the sensor path.

**Figure 4:**
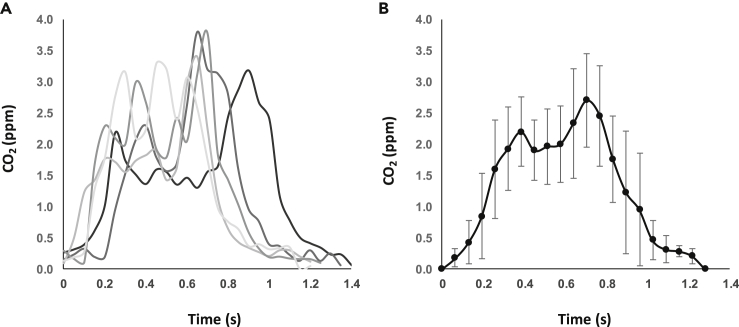
CO_2_ concentration over time for 5 consecutive breaths in a tippler pigeon stationary on a perch in a wind tunnel with an air speed of 7 m s^−1^ (A) Raw data sampled at 20 Hz. (B) Means ± SD every 20^th^ of a breath.

In rested pigeons, CO_2_ signatures were typically M-shaped (Figures 3C and 4), with the second of the two peaks often being the greatest. Here, the rate of change in CO_2_ was typically greater leading up to the second CO_2_ peak, compared with the first, and lowest during the decline following the second peak. In rested pigeons, there were short plateaus in CO_2_ concentration between exhalations that were equal to baseline measurements. In contrast, immediately post-handling, and post-exercise, or in smaller birds, the waveform had only a single peak, lacked plateaus between exhalations, and concentration minima exceeded background CO_2_ concentrations.

### Animal state and respiratory physiology

**Figure 5:**
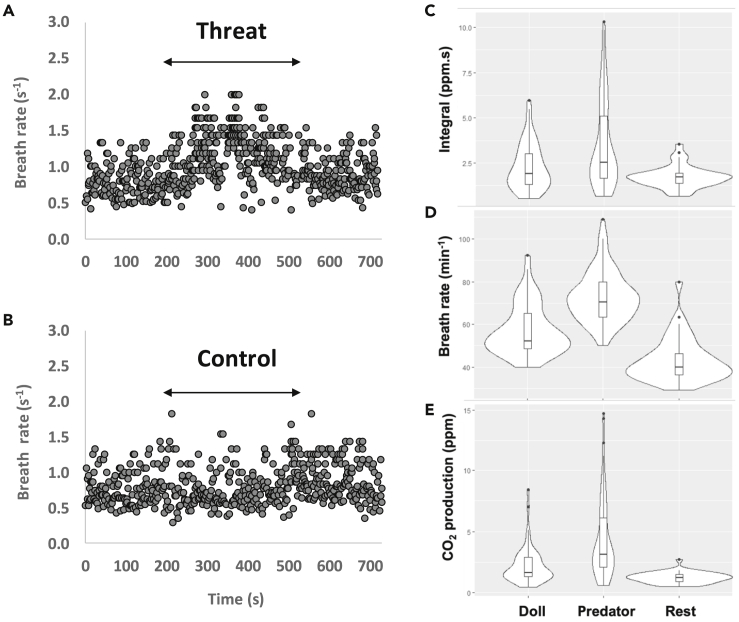
A homing pigeon’s breathing parameters in response to a perceived threat, control novel object, and at rest (A) Changes in respiration rate of a rested individual exposed to a stuffed buzzard and (B) a control novel object (doll), where data points represent single breaths and arrows indicate the duration of exposure to the stimulus. Associated changes in the (C) integral of each breath, (D) breath rate, and (E) CO_2_ production (the product of the breath rate and integral) for 1 min of data per condition with boxplots demonstrating the median, inter-quartile ranges, minimum and maximum (see Tables S1 and S2 for statistical results).

In tippler and homing pigeons, we observed within-individual responses to different treatments. Respiration rates and, in most instances, CO_2_ production, increased during the period of exposure to a stuffed buzzard relative to a rested state, whereas either no response or a smaller response was observed when presented with a control novel object, a doll (e.g., Figure 5, see Tables S1 and S2 for statistical results for homing and tippler pigeons, respectively).

**Figure 6:**
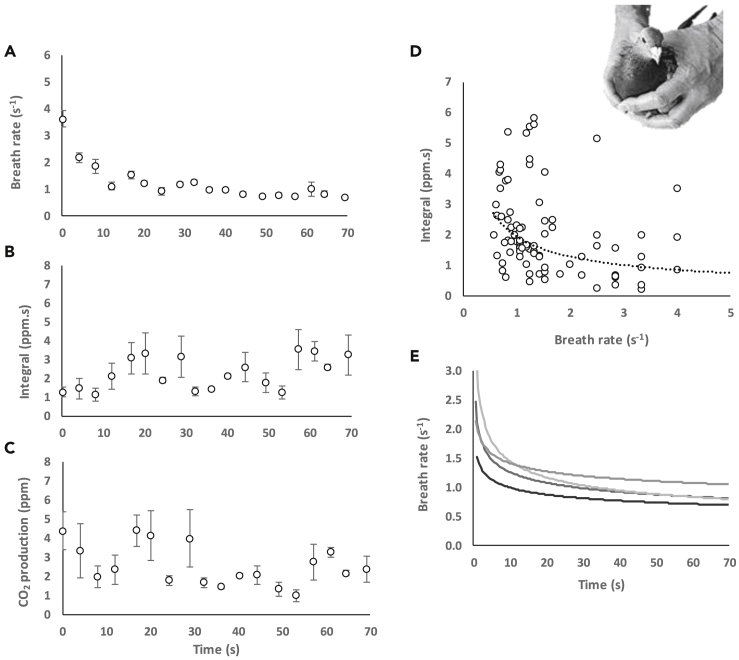
Recovery posthandling and introduction to the tunnel (A) Respiration rate decreased to resting values within 1 min. (B) The integral increased over that minute. (C) CO_2_ production decreased over a minute. Data points for (A–C) are means ± S.E over 4 s for a single tippler pigeon. (D) Total CO_2_ concentration measured per breath increases with decreasing breath rate. (E) Individual variation in breath rate over time. Each line is the best fit for an individual tippler pigeon.

Immediately after handling, breathing rates in tippler pigeons ranged from 2-6 breaths s^−1^. This decreased to 0.6–1 breaths s^−1^ within 1 min, with most of the decline occurring within the first 10 s (Figure 6A). The integral under the exhalation peaks increased over time, although this was highly variable (Figure 6B), and our proxy for CO_2_ production (breathing frequency x integral) decreased (Figure 6C). A negative curvilinear relationship was observed between the total CO_2_ concentration measured per breath and breath rate (Figure 6D). Figure 6E shows inter-individual variation in breath rate over time post-handling.

**Figure 7:**
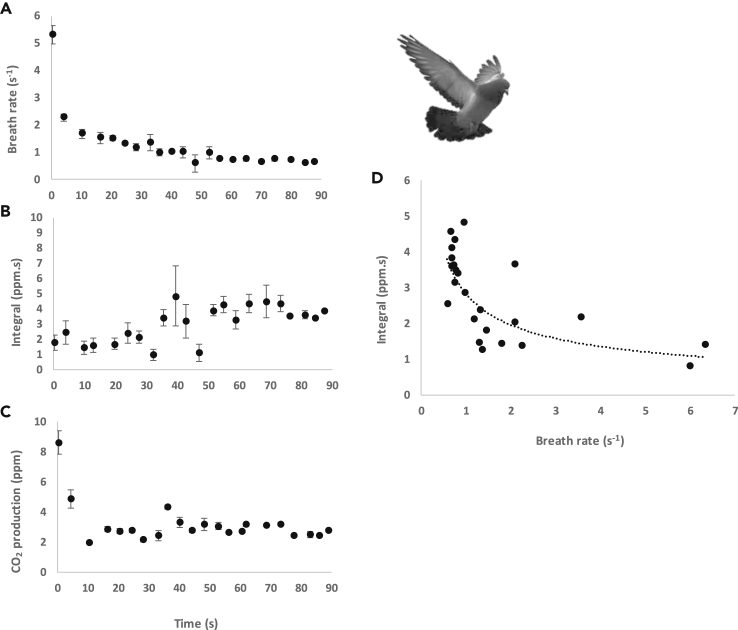
Recovery of a homing pigeon after 10 min of flight at 10 m s^−1^ (A) Breath rate (s^−1^), (B) integral under the exhalation peak (ppm.s), (C) CO_2_ production (ppm), (D) integral versus breath rate. Data points represent a mean over 4 s, and error bars in A–C are ±S.E.

In an example of post-flight recovery, breath rate declined from a maximum of 5.3 breaths s^−1^ to a minimum of 1 breath s^−1^ within 1 min (Figure 7A). The integral of the exhalation peaks increased gradually with recovery time (Figure 7B), whereas CO_2_ production decreased rapidly (within 10 s) (Figure 7C). In all examples, there was a negative correlation between the integral and breath rate post-flight (Figure 6D).

## Discussion

There is abundant literature on how amniote breathing frequency, together with tidal volume, is modulated to meet metabolic demands (Hallam and Dawson, 1993; Kohin et al., 1999); on its uses in the measurement of stress responses (Greenacre and Lusby, 2004; Fucikova et al., 2009; Torné-Noguera et al., 2013; Doss and Mans, 2016, 2017; Liang et al., 2018); its involvement in temperature regulation (El Hadi and Sykes, 1982; Brent et al., 1984; Bucher and Bartholomew, 1986); entering, and arousing from, torpor (Withers, 1977); and how it scales with body mass (Frappell and Baudinette, 1995; Frappell et al., 2001; Mortola and Seguin, 2009). Here, we demonstrate that a NoDIS system can be used to quantify real-time changes in respiration rate with breath-by-breath resolution when the sensor is positioned in the wake of an animal, rather than integrated into a mask or alternative system that requires restraint or tethering (Butler et al., 1977; Franz and Goller, 2003; Wilson et al., 2019). In fact, the system is so sensitive that, for birds as large as pigeons, two sub-peaks in CO_2_ were evident within each exhalation signature, suggesting the anterior and posterior air sacs of the respiratory system empty slightly out of phase with one another (Bretz and Schmidt-Nielsen, 1971; Maina, 2005; Perry et al., 2019). Furthermore, by multiplying the frequency by the integral under the CO_2_ exhalation signature to provide a proxy for CO_2_ production, we were able to document responses to, and recovery from, stressors or exercise over fine-scales (cf. (Franz and Goller, 2003)).

### Limitations in detection of CO_2_ downwind of a constant CO_2_ source

The performance of the system depends on there being discernible pulses in CO_2_, and here we describe the operational limits for this. The NoDIS system that we used is reported to have an RMS noise of 0.16 ppm, which we could confirm with our baseline measurements, and clear signals from the birds (Figure 3). The variability that we obtained in our CO_2_ signals in our calibration trials (Figure 2) was due to inconsistencies in both the rate of expulsion of the CO_2_ and the air flow (although the wind tunnel had a high degree of laminar flow) and increased with the magnitude of the signal.

As predicted, varying wind speed affected the measured CO_2_ concentrations according to the position of the NoDIS sensor relative to the gas source and the rate of gas emission. The detection limits of the sensor (Figure 2) indicate that for gas emission rates of 1 L min^−1^ measurements can be made up to 50 cm immediately downwind of the source if the wind speed is 1 m s^−1^ but this reduces to 10 cm at wind speeds 10 m s^−1^. The detectable CO_2_ wake transect widths in these instances are 10 and 5 cm, respectively, indicating angles of 5.71° and 14.04° either side of a perpendicular line from the source to the sensor. With half the gas emission rate at 0.5 L min^−1^, transect widths become 6 and 4 cm and angles become 3.43° and 11.31°.

These calibration figures illustrate the parameters that influence signal strength and noise. However, beyond these calibrations, when collecting data from animals, further variability in the CO_2_ signal will occur due to (1) movement of the animal (effectively displacing the CO_2_ source relative to the NoDIS sensor), (2) the pulsed nature of CO_2_ expiration during breathing, and (3) the air flow variability itself (direction and speed) if the system is used outside.

### Limitations in detection of CO_2_ downwind of an animal-based CO_2_ source

Animal movement is a major factor in modulating the CO_2_ signal, and we suggest that this can be broadly broken down into three categories: whole body movement (where the animal moves from one site to another), body rotation (where the body turns about its vertical axis only), and head movement only. We recommend that researchers using NoDIS systems film their study animal if possible so that the role of movement can be ascertained. Clearly, for an animal that moves laterally, such as a bird on a perch, thereby displacing the CO_2_ source, this can result in the sensor operating outside the detection plume. In fact, such animal movement would preclude our approach for many animals for much of their time.

Complementary to the methodology demonstrated here, however, there are situations where animals may remain immobile, or at least stay in one spot for extended periods—for example, from incubating (Gabrielsen et al., 1991), sleeping (Tillmann, 2009), or torpid (Nowack et al., 2017) animals, to basking reptiles (Mukherjee et al., 2018), to birds such as flycatchers (Muscicapidae) and raptors that may regularly use particular look-out posts as vantage points (Fitzpatrick, 1980), and to territorial birds singing (Odom et al., 2014). Furthermore, in a manner similar to our pigeons (Figure 7), the method could be used to measure recovery in animals that have engaged in movement outside the sample area before returning. Birds flying back to their nest would be an obvious example.

Body rotation is expected to change the CO_2_ signal in a predictable manner because it effectively results in either a lateral displacement of the CO_2_ source (e.g., if an incubating bird facing upwind rotates 90°) and/or changes the distance between source and sensor (if the bird rotates 180°), both of which have nominally predictable effects on the concentration of the CO_2_ reaching the sensor if properly calibrated. Head movement, especially in long-necked study animals such as swans, can obviously lead to appreciable CO_2_ source displacement, and the extent to which this changes the signal will depend greatly on the situation. A resting swan, for example, is predicted to have an expiration emission rate that is some 7.5 times that of a pigeon (Frappell and Baudinette, 1995) that may make an immediately downwind, but distanced, NoDIS sensor, less sensitive per cm degree of lateral movement (see the extent of the flat top to the distribution in Figure 2) than it would be in a pigeon, although, of course, the pigeon would move its head absolutely less.

The carefully controlled wind conditions of the wind tunnel standardize an important element of the protocol, and the value of the CO_2_ concentration data over time in the wild will be critically dependent on the variability in wind speed and the turbulence. Variation in wind speed (gustiness) increases with overall mean wind speed, so the CO_2_ pulses detected by the NoDIS system will vary accordingly, specifically having the period of the exhalent pulse contracted or expanded, with accompanying changes in pulse height. Overall, this should not change respiration rate values measured over a number of cycles, but it will alter interpretation of patterns of air exhalation (see below) and may alter the values of the integrals of the CO_2_ concentration under the expiration pulse (see below), although expanded exhalation periods should be accompanied by decreased CO_2_ production. These issues may be largely mitigated by having high-resolution measurement of wind speed (and vector) at the site so that, if necessary, corrections could be applied or at least data filtered to exclude aberrant gusts or periods of calm.

Another option, for specific cases where there is no wind, is to blow air at an appropriate rate past the study animal. This is easiest for animals in prescribed hollows, such as bats in their roosting boxes, but may also work for animals outside. However, this has previously been done successfully in a number of studies using standard respirometry and will not necessarily alter the subject’s metabolic rate (e.g., Nowack et al., 2020). The environmental conditions and silent excurrent flow generators in these studies could be used as a starting point for exploring how we can take the temporal resolution of wake respirometry to the field.

### Wake respirometry: Future developments

Given the accuracy of the NoDIS system in measuring CO_2_ concentrations in precise locations downwind of the study animal, it is tantalizing to speculate whether this approach might enable researchers to determine metabolic rate in free-living animals. Effectively, the integral of every CO_2_ pulse should be proportional to the sum of the CO_2_ expired in that exhalation and should indicate relative changes in metabolic rate.

Derivation of total CO_2_ emission for determining absolute metabolic rate is more challenging. Ideally, a means to capture a full transect of the wake is required, which could be achieved by moving the sensor closer to the study animal or working with a greater NoDIS emitter distance. It might be possible to use a cone of equal or greater width compared with the CO_2_ wake to ensure that all exhaled CO_2_ passes a single NoDIS sensor for integration of the downstream wake. Alternatively, a series of inhalant tubes could sample the wake and mix the air for analysis by a single NoDIS sensor. Or data from multiple NoDIS sensors within the CO_2_ footprint could be integrated and summed to provide the complete CO_2_ footprint per exhalation and over longer time periods. Furthermore, the open-path sensor used in this study, with its high temporal resolution (20 Hz), could be used as an auxially input reference to a dual cell closed-path CO_2_ analyzer (e.g. LI-7000) samping gases from upsteam and downstream of an animal to allow rapid continuous calculation of ΔCO_2_. In any case, some sort of calibration would be desirable but simulation of CO_2_ emissions from the study animal at different ventilation frequencies on site *post hoc* by bleeding gas from models may help. Furthermore, CO_2_ flux (μmol m^−2^ s^−1^), and subsequently, metabolic rate, could be calculated using the eddy covariance method, which requires sampling air speed and direction in tandem.

Overall, this work has demonstrated that the new, portable generation of CO_2_ sensors can provide insight into stress and respiratory patterns in unrestrained animals and, as a result, that they could be used to document stress responses in wild animals. With suitable consideration of the limitations imposed by factors such as animal movement and wind variability, wake respirometry should have a future that will help with a diverse suite of issues, such as determination of the extent to which urbanization (Charmantier et al., 2017), ecotourism (Mullner et al., 2004), changing temperatures, or natural disasters (Nowack et al., 2017) might affect target animals and indeed, comparison of the effects of different stressors (Clinchy et al., 2013). Beyond that, it also opens the way for potential measurements of the metabolic rate of unrestrained birds, both resting, and in the case of wind tunnels, in flight.

### Limitations of the study

Although our wake respirometry method enables respiration rate, on a breath-by-breath basis, to be assessed and linked to stress and energetics by examining CO_2_ exhalation signatures over time emitted by unrestrained animals, our approach needs further refinement to allow metabolic rate to be calculated. Specifically, CO_2_ concentrations across the full CO_2_ wake must be measured or modeled based on specific point values. The factors limiting measurement of a complete CO_2_ wake transect include the following: (1) the sensor emitter width should be substantially narrower than the wake width so that a defined fraction of the wake’s width is sampled, and (2) the movement of the animal (head/body rotation or full body displacement) can be incorporated into calculations because this redirects the wake relative to the sensor. Movement of animal subjects will not influence breathing frequency calculations as long as exhalations are not completely missed by the sensor, but measurement of overall CO_2_ production will be variable. Future research can refine set-ups to direct (e.g., funnel) complete exhalations across a single or multiple sensors or apply the eddy covariance method using measurements of wind speed and direction to calculate CO_2_ flux (μmol m^−2^ s^−1^), which should allow subsequent calculation of metabolic rate.

## STAR★Methods

### Key resources table

**Table:** 

REAGENT or RESOURCE	SOURCE	IDENTIFIER
**Deposited data**		

Example data for recovery post handling	Figshare	10.6084/m9.figshare.19566046
Example data for recovery post flight	Figshare	10.6084/m9.figshare.19566028
Respiratory responses of pigeons to different stimuli	Figshare	10.6084/m9.figshare.19566013
Example CO_2_ exhalation signatures from zebra finch, starling and pigeon	Figshare	10.6084/m9.figshare.20632914
Calibration data	Figshare	10.6084/m9.figshare.20632947

**Experimental models: Organisms/strains**		

Zebra finch	Max Planck Institute for Ornithology, Seewiesen	Max Planck Institute for Ornithology, Seewiesen
Starling	Max Planck Institute for Ornithology, Seewiesen	Max Planck Institute for Ornithology, Seewiesen
Tippler pigeons	Max Planck Institute for Ornithology, Seewiesen	Max Planck Institute for Ornithology, Seewiesen
Homing pigeons	Max Planck Institute for Ornithology, Seewiesen	Max Planck Institute for Ornithology, Seewiesen
Homing pigeon	Swansea University	Swansea University

**Software and algorithms**		

OriginLab	OriginLab Corporation	https://www.originlab.com
R	R Core Team, 2021	https://www.R-project.org

### Resource availability

#### Lead contact

Further information and requests regarding the methods or data used should be directed to the lead contact, Kayleigh A. R. Rose (k.a.r.rose@swansea.ac.uk).

#### Materials availability

This study did not generate new unique reagents.

### Experimental model and subject details

#### Captive birds used in wind tunnel trials

Data were recorded from adult captive tippler pigeons (*ca*. 300 g, 2 male, 2 female), homing pigeons (n = 5, *ca*. 400 g, 2 male, 3 female), a starling (*ca.* 70 g, male) and a zebra finch (*ca*. 10 g, male) in a closed system wind tunnel at the Max Planck Institute for Biological Intelligence, Germany. Birds were kept in aviaries beside a closed system wind tunnel according to §11 Permission (§11 TierSchG) and were accustomed to being inside the tunnel for flight training. Animal experiments performed in Seewiesen, Germany, were conducted according to the regulations of the government of Upper Bavaria (Germany protocol numbers: AZ 55.2-1-54-2532-86-2015; 311.5-5682.1/1-2014-021). At Swansea University, data were recorded from a hand-reared homing pigeon (female, *ca*. 350 g) accustomed to flight training in the open jet wind tunnel. Here, homing pigeons were housed in an outdoor loft with aviary under an establishment licence. Experiments were carried out under the project licence (X5770C662) and ethical permission for this work was given by Swansea University AWERB (200418/65).

### Methods details

#### Definition of the CO_2_ shadow downwind of a source

##### Apparatus

Non-dispersive infrared spectroscopy uses infrared radiation that is emitted across an (open) path of defined length, across which the CO_2_ is to be measured, with the radiation being detected at the distal end of the path by a lead selenide sensor. Both water vapour and CO_2_ absorb the radiation so gas densities can be determined by considering the absorption with respect to a reference. We used the LI-7500A Open Path CO_2_/H_2_O Analyzer (Lincoln, Nebraska, USA), which has an emitter-sensor distance of 125 mm, resolution 0.01 ppm, and error within 1% of reading. During our use of this system, we deployed it in a vertical orientation and set it to sample at 20 Hz (RMS noise 0.16 ppm at 370 ppm CO_2_).

##### Calibrations

We assessed the viability of our approach by conducting trials under varying conditions. We used a defined gas mix of 4% CO_2_/air (BOC) to carry out our calibrations in an open jet style wind tunnel custom designed for bird flight (test section width 1.8 m, length 2.2 m, height 1.5 m) in Swansea University, UK. Rubber tubing (10 mm outer diameter) and a variable flow metre were used to release the gas mix at two fixed flow rates (0.5 and 1 L min^−1^), via a metal tube inserted through the ceiling of the tunnel, which extended to a central position 60 cm inside the test section. The NoDIS sensor was positioned on a stand at the same height as the source to record CO_2_ (ppm) at three source-sensor distances (10, 30, 50 cm) and three wind speeds (1, 5 and 10 m s^−1^). At each emission rate, windspeed, and distance (d) from the source, ten seconds of CO_2_ (ppm) data was logged (20 Hz) every 5 or 2.5 cm along the wake transect until the signal was no longer detectable.

#### CO_2_ signals from captive birds in different states

We investigated whether our set-up could detect responses of the tippler and homing pigeons to the following treatments; i) release after being handled, which was assessed by keeping birds in a darkened box before they were held by an experimenter for 2 minutes and then introduced to the tunnel perch, and proximity to ii) a potential threat (stuffed buzzard *Buteo buteo*) and iii) a similar-sized control novel object (rag doll). Within-individual responses to the buzzard and doll were assessed when the birds were in a rested state on the perch.

The perch (55 cm tall, 8 cm wide) was positioned in the centre of the test section while the NoDIS was fixed to a stand positioned 46 cm downstream. The perch width was relatively small to prevent birds moving to the left or right relative to the sensor. Birds always faced into the wind when the tunnel was on. Fixed windspeeds (7 and 10 m s^−1^ for tippler and homing pigeons, respectively) were chosen to ensure a clear signal with minimal variation in CO_2_ concentration between exhalations.

The wind tunnel room was 17-19°C. Lights in the study room were dimmed to a low level and noise additional to that of the wind tunnel was kept to a minimum. Two experimenters were in the room during all trials, which were conducted during normal active hours. A typical trial consisted of a bird being placed inside a darkened carrier box (dimensions 50 × 35 × 30 cm) for 10 minutes in the wind tunnel room. The bird was then removed from the box and held for 2 minutes with fingers around both sides of the body. The same experimenter restrained all birds (Rabdeau et al., 2019). At the same time, a baseline CO_2_ trace was recorded in the absence of the bird with the tunnel on. The bird was then placed on the perch upstream of the sensor and left to sit quietly. After 20 min, either the taxidermy buzzard or rag doll was presented outside the test section and upstream of the study bird and held there for 2 minutes. Another 20 minutes of quiet time followed before the second stimulus was presented. The order in which the stimuli were introduced within trials was randomised and birds were presented with each stimulus only once to avoid habituation.

For a comparison of the CO_2_ exhalation signal between birds of different body size at a single wind speed and bird-sensor distance (46 cm and 2 m s^−1^), resting data were collected from a perched tippler pigeon, starling, and zebra finch in the Max Planck wind tunnel. Twelve starlings were flown as a flock at 10 m s^−1^ for 10 minutes as part of their usual training regime, after which, one individual was kept within the tunnel to recover on a perch. Similarly, ten zebra finches were flown as a flock at 8 m s^−1^ for 10 minutes, following which one individual was kept within the tunnel for breath recordings.

Data were also collected behind a homing pigeon in the test section of the tunnel at Swansea University after 11 minutes of flight training at 10 m s^−1^ for post-flight recovery data. The bird was perched 20 cm upstream of the NoDIS (perch height 60 cm) and the wind speed remained at 10 m s^−1^ during data collection after the flight training. Lights were dimmed at the end of a training session for respirometry measurement and experimental temperatures ranged from 18.8–19.2°C.

### Quantification and statistical analysis

#### Data processing and extraction

CO_2_ data (ppm) were corrected for baseline drift in OriginLab 2021 using linear interpolation between baseline data collected in the absence of a bird at the beginning and end of trials or at regular intervals throughout calibration experiments in the absence of CO_2_ emission. Calibration measurements, exhalation and breath cycle parameters were examined using in house software (DDMT, Wildbyte Technologies, http://wildbytetechnologies.com). Data for CO_2_ over time were isolated for each expiration from drift-corrected and baseline-corrected data. Breathing frequency (breaths per unit time) was calculated as the reciprocal of the breath cycle period. A proxy for CO_2_ production was calculated by multiplying breathing frequency by the integral of the CO_2_ signal.

#### Statistical analyses

Statistical analyses were conducted in R Studio using R version 4.0.3 (R Development Core Team 2019). Non-parametric Kruskal-Wallis and Dunn (holm adjusted) post-hoc tests were conducted to investigate within-individual differences in pigeon breath rate, integral of the CO_2_ signature and CO_2_ production (breath rate x integral) under three different conditions: in a rested and undisturbed state; when exposed to a control novel object (rag doll); and when exposed to a potential threat (stuffed buzzard). In all cases, assumptions for parametric one-way ANOVAs were not met and this was confirmed by examining qq plots and histograms of model standardised residuals as well as Shapiro Wilk tests to confirm a distribution significantly different from normal. For each pigeon, one minute of breath-by-breath data was used per condition. Data were investigated separately for each pigeon because of the variability of signal strength that is expected due to each bird’s varying body form and head movement, our small sample size, and additional variation in measurements expected due to individual differences in body mass.

## Data Availability

The raw datasets for figures in the paper are available here (Figshare: 10.6084/m9.figshare.19566046, 10.6084/m9.figshare.19566028, 10.6084/m9.figshare.19566013, 10.6084/m9.figshare.20632914, 10.6084/m9.figshare.20632947). This paper does not report original code. Any additional information required to reanalyze the data reported in this paper is available from the lead contact upon request.
