# Supplementary material for: Wake respirometry allows breath-by-breath assessment of ventilation and CO2 production in unrestrained animals
Source: iScience. 2022 Aug 14;25(9):104878. doi: 10.1016/j.isci.2022.104878 (PMC9437847; doi:10.1016/j.isci.2022.104878)
Supplement: Document S1. Tables S1 and S2 [file mmc1.pdf]

iScience, Volume *25*

Supplemental information

Wake respirometry allows breath-by-breath assessment of ventilation and CO_2 production in unrestrained animals

Kayleigh A.R. Rose, Rory P. Wilson, Claudia Ramenda, Hermina Robotka, Martin Wikelski, and Emily L.C. Shepard

Table S1. Results of Kruskal-Wallis tests and Dunn (holm adjusted) post hoc tests to investigate differences in the respiratory parameters of individual homing pigeons exposed to different stimuli, related to Figure 5.

ID	Parameter	Median	e^2	X^2 (df)	P	n	Comparison	z	P.unaj	P.adj
HP1<br>female	Integral<br>(ppm.s)	Rest: 1.72<br>Doll: 1.92<br>Predator: 2.52	0.089	16.71 (2)	**0.0002**	169	doll - predator<br>doll - rest<br>predator - rest	-1.47<br>0.70<br>2.43	0.142<br>0.486<br>0.015	0.283<br>0.486<br>**0.046**
	Breath rate<br>(/min)	Rest: 40<br>Doll: 52.17<br>Predator: 70.59	0.555	94.19 (2)	**<0.001**	169	doll - predator<br>doll - rest<br>predator - rest	-5.62<br>4.09<br>9.44	<0.001<br><0.001<br><0.001	**<0.001**<br>**<0.001**<br>**<0.001**
	CO_2<br>production<br>(ppm)	Rest: 1.24<br>Doll: 1.65<br>Predator: 3.14	0.310	53.49 (2)	**<0.001**	169	doll - predator<br>doll - rest<br>predator - rest	-4.58<br>1.73<br>7.10	<0.001<br>0.083<br><0.001	**<0.001**<br>0.083<br>**<0.001**
HP2<br>male	Integral<br>(ppm.s)	Rest: 1.71<br>Doll: 1.47<br>Predator: 1.94	n/a	3.26 (2)	0.196	219	n/a	n/a	n/a	n/a
	Breath rate<br>(/min)	Rest: 40<br>Doll: 50<br>Predator: 63	0.183	41.52 (2)	**<0.001**	219	doll - predator<br>doll - rest<br>predator - rest	-6.44<br>-3.84<br>3.42	<0.001<br><0.001<br><0.001	**<0.001**<br>**<0.001**<br>**<0.001**
	CO_2<br>production<br>(ppm)	Rest: 1.76<br>Doll: 1.23<br>Predator: 2.00	0.037	10.06 (2)	**0.007**	219	doll - predator<br>doll - rest<br>predator - rest	-2.74<br>-2.88<br>0.18	0.006<br>0.004<br>0.854	**0.012**<br>**0.011**<br>0.854
HP3<br>female	Integral<br>(ppm.s)	Rest: 2.18<br>Doll: 2.80<br>Predator: 2.39	0.035	8.38 (2)	**0.016**	184	doll - predator<br>doll - rest<br>predator - rest	2.57<br>2.66<br>-0.10	0.010<br>0.007<br>0.918	**0.020**<br>**0.023**<br>0.918
	Breath rate<br>(/min)	Rest: 41.38<br>Doll: 40<br>Predator: 57.14	0.440	81.61 (2)	**<0.001**	184	doll - predator<br>doll - rest<br>predator - rest	-7.84<br>-1.53<br>7.74	<0001<br>0.126<br><0.001	**<0.001**<br>0.126<br>**<0.001**
	CO_2<br>production<br>(ppm)	Rest: 1.53<br>Doll: 1.88<br>Predator: 1.95	0.045	10.22 (2)	**0.006**	184	doll - predator<br>doll - rest<br>predator - rest	-0.78<br>1.89<br>3.08	0.435<br>0.058<br>0.002	0.435<br>0.117<br>**0.006**
HP4<br>male	Integral<br>(ppm.s)	Rest: 2.67<br>Doll: 3.46<br>Predator: 3.98	0.054	11.27 (2)	**0.003**	174	doll - predator<br>doll - rest<br>predator - rest	-1.43<br>1.72<br>3.32	0.154<br>0.087<br><0.001	0.154<br>0.173<br>**0.003**
	Breath rate<br>(/min)	Rest: 41.28<br>Doll: 46.15<br>Predator: 46.15	0.195	35.30 (2)	**<0.001**	174	doll - predator<br>doll - rest<br>predator - rest	0.98<br>5.40<br>4.30	0.033<br><0.001<br><0.001	**0.033**<br>**<0.001**<br>**<0.001**

	CO_2 production (ppm)	Rest: 1.75<br>Doll: 2.60<br>Predator: 2.76	0.140	25.86 (2)	**<0.001**	174	doll - predator<br>doll - rest<br>predator - rest	-0.78<br>3.72<br>4.60	0.435<br><0.001<br><0.001	0.435<br>**<0.001**<br>**<0.001**

Table S2. Results of Kruskal-Wallis tests and Dunn (holm adjusted) post hoc tests to investigate differences in the respiratory parameters of individual tippler pigeons exposed to different stimuli, related to Figure 5.

ID	Parameter	Median	e^2	X^2 (df)	P	n	Comparison	z	P.unaj	P.adj
Emil<br>male	Integral	Rest: 2.68<br>Predator: 2.08	0.065	6.90 (1)	**0.009**	93	Rest-predator only	n/a	n/a	n/a
	Breath rate	Rest: 38.11<br>Predator: 54.55	P	51.91 (1)	**<0.001**	93	Rest-predator only	n/a	n/a	n/a
	CO_2 production	Rest: 1.58<br>Predator: 1.86	0.034	4.13 (1)	**0.042**	93	Rest-predator only	n/a	n/a	n/a
Emils_son_2<br>male	Integral	Rest: 3.52<br>Doll: 2.91<br>Predator: 1.77	0.199	30.22 (2)	**<0.001**	145	doll - predator<br>doll - rest<br>predator - rest	3.35<br>-2.13<br>-5.44	<0.001<br>0.033<br><0.001	**0.002**<br>**0.033**<br>**<0.001**
	Breath rate	Rest: 44.44<br>Doll: 52.17<br>Predator: 47.08	n/a	1.91 (2)	0.385	145	n/a	n/a	n/a	n/a
	CO_2 production	Rest: 2.73<br>Doll: 2.25<br>Predator 1.35	0.018	26.95 (2)	**<0.001**	145	doll - predator<br>doll - rest<br>predator - rest	3.35<br>-1.79<br>-5.09	<0.001<br>0.073<br><0.001	**0.002**<br>0.071<br>**<0.001**
TF1<br>female	Integral	Rest: 5.15<br>Doll: 3.66<br>Predator: 4.60	0.159	19.29 (2)	**<0.001**	112	doll - predator<br>doll - rest<br>predator - rest	-2.47<br>-4.37<br>-2.08	0.013<br><0.001<br>0.038	**0.027**<br>**<0.001**<br>**0.038**
	Breath rate	Rest: 31.18<br>Doll: 36.36<br>Predator: 41.38	0.304	35.13 (2)	**<0.001**	112	doll - predator<br>doll - rest<br>predator - rest	-2.47<br>-4.37<br>-2.08	0.014<br><0.001<br>0.038	**0.027**<br>**<0.001**<br>**0.038**
	CO_2 production	Rest: 2.74<br>Doll: 2.22<br>Predator: 3.03	0.063	8.91 (2)	**0.012**	112	doll - predator<br>doll - rest<br>predator - rest	-2.98<br>-1.61<br>1.20	0.003<br>0.108<br>0.230	**0.008**<br>0.216<br>0.230

TF2<br>female	Integral	Rest: 1.72<br>Doll: 1.34<br>Predator: 2.18	0.070	13.95 (2)	**<0.001**	174	doll - predator<br>doll - rest<br>predator - rest	-3.73<br>-1.94<br>1.59	<0.001<br>0.053<br>0.112	**<0.001**<br>0.106<br>0.112
	Breath rate	Rest: 43.48<br>Doll: 50<br>Predator: 50	0.126	23.55 (2)	**<0.001**	174	doll - predator<br>doll - rest<br>predator - rest	0.30<br>4.42<br>4.11	0.767<br><0.001<br><0.001	0.767<br>**<0.001**<br>**<0.001**
	CO_2 production	Rest: 1.35<br>Doll: 1.12<br>Predator: 1.73	0.077	15.18 (2)	**<0.001**	174	doll - predator<br>doll - rest<br>predator - rest	-3.73<br>-0.76<br>2.76	<0.001<br>0.447<br>0.006	**<0001**<br>0.447<br>**0.011**
TF3<br>Female	Integral	Rest: 2.95<br>Doll: 3.21<br>Predator: 3.64	n/a	1.33 (2)	0.515	142	n/a	n/a	n/a	n/a
	Breath rate	Rest: 45.15<br>Doll: 41.28<br>Predator: 50	0.210	31.13 (2)	**<0.001**	142	doll - predator<br>doll - rest<br>predator - rest	-5.60<br>-2.79<br>2.83	<0.001<br>0.005<br>0.005	**<0.001**<br>**0.005**<br>**0.009**
